# O.4.5-10 Self-control and self-efficacy positively influence physical activity levels

**DOI:** 10.1093/eurpub/ckad133.218

**Published:** 2023-09-11

**Authors:** Radoslawa Herzog-Krzywoszanska, Wiktor Potoczny, Lukasz Krzywoszanski

**Affiliations:** Pedagogical University of Krakow, Poland; radoslawa.herzog-krzywoszanska@up.krakow.pl; Pedagogical University of Krakow, Poland; radoslawa.herzog-krzywoszanska@up.krakow.pl; Pedagogical University of Krakow, Poland; radoslawa.herzog-krzywoszanska@up.krakow.pl

## Abstract

**Purpose:**

Physical activity is essential for maintaining good health and preventing chronic diseases such as obesity, cardiovascular disease, and type 2 diabetes. Understanding the factors that influence physical activity can help to design interventions that promote physical activity and prevent these diseases.The purpose of this study was to investigate the relationship between self-control, self-efficacy, and physical activity.

**Methods:**

The study included 375 participants between the ages of 19 and 43 who completed the Brief Self-Control Scale (BSCS), Generalized Self-Efficacy Scale (GSES), and a physical activity inventory. A linear regression model was used to analyze the relationships between self-control and physical activity, with self-efficacy as a mediating variable.

**Results:**

The analysis of the data showed that self-control positively correlated with the number of hours per week devoted to physical activity. The results also indicated that self-efficacy mediated this relationship. Specifically, higher levels of self-control were associated with higher levels of self-efficacy, which in turn were associated with higher levels of physical activity.

**Conclusions:**

The findings of this study highlight the importance of both self-control and self-efficacy in promoting physical activity. Improving self-control and self-efficacy may be effective strategies for increasing physical activity levels.

